# Erythrocyte membrane fatty acid fluidity and risk of type 2 diabetes in the EPIC-Potsdam study

**DOI:** 10.1007/s00125-014-3421-7

**Published:** 2014-10-25

**Authors:** Janine Kröger, Simone Jacobs, Eugène H. J. M. Jansen, Andreas Fritsche, Heiner Boeing, Matthias B. Schulze

**Affiliations:** 1 Department of Molecular Epidemiology, German Institute of Human Nutrition Potsdam-Rehbrücke, Arthur-Scheunert-Allee 114-116, 14558 Nuthetal, Germany; 2 Center for Health Protection Research, National Institute for Public Health and the Environment, Bilthoven, the Netherlands; 3 Department of Internal Medicine, Division of Endocrinology, Diabetology, Nephrology, Vascular Disease, and Clinical Chemistry, University Hospital of the Eberhard Karls University, Tübingen, Germany; 4 Institute for Diabetes Research and Metabolic Diseases of the Helmholz Centre Munich at the University of Tübingen (IDM), Partner in the German Center for Diabetes Research (DZD), Tübingen, Germany; 5 Department of Epidemiology, German Institute of Human Nutrition Potsdam-Rehbrücke, Nuthetal, Germany

**Keywords:** Erythrocyte membrane, Fatty acids, Incidence, Lipophilic index, Melting point, Prospective studies, Type 2 diabetes mellitus

## Abstract

**Aims/hypothesis:**

The fluidity of cell membranes has been hypothesised as an important link in the association of fatty acids (FAs) with diabetes risk. The lipophilic index, which can be derived from the FA profile of blood or tissues, has recently been proposed as a novel measure of cell membrane FA fluidity. In this study we aimed to evaluate the lipophilic index in relation to the incidence of type 2 diabetes.

**Methods:**

We applied a nested case-cohort design (*n* = 1,740, including 362 cases) within the EPIC-Potsdam study, which involves 27,548 middle-aged men and women. Erythrocyte membrane FA proportions were measured at baseline and physician-confirmed incident diabetes was assessed during a mean follow-up of 7.0 years. The lipophilic index was calculated as the sum of the products of the FA proportions with the respective FA melting points.

**Results:**

After multivariable adjustments, including body size measures, there was a positive association between the lipophilic index and diabetes risk (HR comparing top with bottom quartile 1.59 (95% CI 1.08, 2.34), p for trend across quartiles = 0.005). Adjustment for FAs, which are considered established diabetes risk markers, did not substantially attenuate this association.

**Conclusions/interpretation:**

A high lipophilic index, reflecting lower membrane fluidity, may be associated with a higher risk of type 2 diabetes. Our data corroborate the hypothesis that membrane fluidity may be an important mediator that links intake and metabolism of FAs to diabetes risk.

**Electronic supplementary material:**

The online version of this article (doi:10.1007/s00125-014-3421-7) contains peer-reviewed but unedited supplementary material, which is available to authorised users.

## Introduction

The fatty acid (FA) profile of blood and tissues integrates the complex interplay between dietary intake of FAs and endogenous FA metabolism, and has been shown to be associated with risk of type 2 diabetes in a number of studies [1–6]. As a potential mechanism, there is a long-held view that the fluidity of the cell membrane, which is importantly determined by its FA composition, affects cellular functions [7]. Despite this notion of an important role of membrane fluidity for diabetes development, there is scarce data to support the relevance of this hypothesis. In cell culture studies, it has been shown that membrane fluidity affects glucose transport across membranes as well as the properties of the insulin receptor [8–10]. However, we are not aware of human studies investigating the relevance of alterations of the fluidity of cell membranes for the incidence of type 2 diabetes.

Membrane fluidity is strongly determined by the van der Waals forces between FAs in the phospholipid bilayers. These van der Waals forces are dependent on the chemical structure of the FA molecules. The longer and straighter the FA chain, the higher the van der Waals forces and the lower the FA fluidity, as reflected by a high FA melting point. Consequently, long-chain saturated FAs (SFAs) with a straight FA chain are characterised by a relatively high melting point, whereas polyunsaturated fatty acids (PUFAs) with a high number of double bonds, leading to a complex three-dimensional structure, generally have lower melting points.

Only recently, the lipophilic index has been proposed as a measure of overall FA fluidity, which can be easily derived from the FA composition of biological tissues. The lipophilic index is characterised as the mean of the melting points of FAs in biological tissues weighted by their specific concentrations [11, 12]. Recent studies have suggested that a higher lipophilic index in plasma and adipose tissue, reflecting decreased FA fluidity, is associated with a higher risk of CHD [11, 12]. However, no significant association was observed for an erythrocyte lipophilic index [11]. We are not aware of studies that have related the lipophilic index to the incidence of type 2 diabetes.

In the current study, we aimed to investigate prospectively the lipophilic index as a measure of the FA fluidity of erythrocyte membranes in relation to the incidence of type 2 diabetes.

## Methods

### Study population

The European Prospective Investigation into Cancer and Nutrition (EPIC)-Potsdam study involves 27,548 participants (16,644 women aged mainly 35–65 years and 10,904 men aged mainly 40–65 years) recruited from the general population in Potsdam, Germany, between 1994 and 1998 [13]. Follow-up questionnaires have been administered every 2–3 years to identify incident cases of type 2 diabetes (response rates 91–96%). In the EPIC-Potsdam cohort, 849 cases of incident type 2 diabetes were identified during a mean follow-up time of 7.0 years (see electronic supplementary material [ESM] Methods for details on ascertainment of type 2 diabetes). Consent was obtained from all participants. Approval was given by the Ethical Committee of the State Brandenburg, Germany.

A case-cohort study [14] within the prospective EPIC-Potsdam study was designed. We randomly selected 2,500 individuals from all participants of the EPIC-Potsdam study providing blood (*n* = 26,444) for a subcohort, of which 1,406 remained after exclusion of participants with any history of diabetes at baseline or with self-reported diabetes during follow-up without physician confirmation (*n* = 120), with missing follow-up information (*n* = 58), with FA concentrations that were considered to be unreliable (*n* = 598, see ESM Methods for details), with missing or implausible biomarker values (*n* = 117, see ESM Methods for details) or with a history of cardiovascular disease or cancer at baseline (*n* = 201). We excluded participants with a history of cardiovascular disease or cancer since these participants may have changed their habitual diet after diagnosis resulting in changed membrane fluidity. Furthermore, it has been suggested that erythrocyte membrane fluidity changes after an acute myocardial infarction event [15]. Of the 849 participants with incident diabetes identified in the full cohort, 362 remained for analyses after excluding cases with missing blood samples (*n* = 48), with FA concentrations that were considered to be unreliable (*n* = 313, see ESM Methods for details), with missing or implausible biomarker values (*n* = 36, see ESM Methods for details) or with a history of cardiovascular disease or cancer at baseline (*n* = 90).

### Measurement of FA composition in erythrocytes and calculation of lipophilic index

Peripheral venous blood samples were taken at the baseline examination and were centrifuged at 1000 *g* for 10 min at 4°C. Plasma, erythrocytes and buffy coat were removed and stored at −80°C. Erythrocyte membrane FAs were measured between February and June 2008. A detailed description of the laboratory methods can be found in the ESM Methods. Briefly, FA methyl esters (FAME) were separated on a GC-3900 gas chromatograph (Varian, Middelburg, the Netherlands) equipped with a 100 m × 0.25 mm ID WCOT-fused silica capillary column and flame ionisation detector with separation of FAME peaks based on mixed FAME standards (Sigma Aldrich, St Louis, MO, USA). The FAs were expressed as the percentage of total FAs present in the chromatogram.

The lipophilic index for the FA composition of the erythrocyte membranes has been defined as a weighted mean of the FA melting points, as proposed in earlier publications [11, 12]. More specifically, the index was calculated as the sum of the products of the proportion (% of total FAs) with the melting point (°C) of each individual FA, divided by the sum of the proportions of all FAs:

$$ \mathrm{Lipophilic}\kern0.15em \mathrm{index}=\frac{{\displaystyle {\sum}_{\kappa}\left[\mathrm{proportion}\kern0.3em \mathrm{of}\kern0.1em \mathrm{F}\mathrm{A}\kern0.1em \left(\%\right)\kern-0.2em \times \kern-0.2em \mathrm{melting}\ \mathrm{point}\ \mathrm{of}\kern0.1em \mathrm{F}\mathrm{A}\kern0.1em \left({}^{\circ}\mathrm{C}\right)i\right]}}{{\displaystyle {\sum}_{\kappa}\left[\mathrm{proportion}\ \mathrm{of}\ \mathrm{F}\mathrm{A}\ \left(\%\right)i\right]}} $$

where *i* represents the individual FA, and *k* represents the number of FAs used to calculate the lipophilic index.

Melting points of FAs were obtained from the LipidBank database (http://lipidbank.jp/, accessed 19 November 2013) in line with a previous publication on the lipophilic index and risk of myocardial infarction [12]. Melting points were not available in the LipidBank database for the following FAs: 16:1 *n*-9, 16:2 *n*-4, 16:3 *n*-4, 20:2 *n*-6, 20:3 *n*-6, 22:2 *n*-6 and 22:4 *n*-6. Hence, these FAs could not be considered in the calculation of the lipophilic index (22% of measured FAs not considered, 78% of measured FAs considered, for creating the lipophilic index). Median proportions of the FAs without information on the melting point in the erythrocyte membranes were generally low in our subcohort (0.10% for 16:1 *n*-9, 0.03% for 16:2 *n*-4, 0.10% for 16:3 *n*-4, 0.25% for 20:2 *n*-6, 1.5% for 20:3 *n*-6, 0.05% for 22:2 *n*-6 and 2.7% for 22:4 *n*-6).

### Measurement of further biomarkers

Measurement of plasma HDL-cholesterol, triacylglycerol, Gamma-glutamyl transferase (GGT), C-reactive protein (CRP), fetuin-A and random glucose as well as HbA_1c_ followed standard procedures [16]. Plasma total adiponectin concentrations were determined by an ELISA (Linco Research, St Charles, MO, USA). All assays were performed according to the manufacturer’s description. Plasma biomarker values were multiplied with a sex-specific correction factor of 1.16 for women and 1.17 for men to take into account the plasma dilution caused by citrate addition [17].

### Other measurements

We used baseline information on dietary intake (assessed with a semi-quantitative food frequency questionnaire [FFQ]), education, smoking and physical activity (assessed with a self-administered questionnaire and a personal computer-guided interview) for our analyses. Details on the assessment of these variables can be found in the ESM Methods.

### Statistical analysis

We performed Cox proportional hazards analysis stratified by age to study the association between the lipophilic index and the hazard of type 2 diabetes. Age was used as the primary time-dependent variable in all models, with entry time defined as the participant’s age at recruitment and exit time as the date of diagnosis of diabetes (either type 2 diabetes [ICD10: E11; www.who.int/classifications/icd/en/] or other types of diabetes mellitus [ICD10: E10, E13, E14] because such participants were no longer at risk of developing type 2 diabetes), death or return of the last follow-up questionnaire. To account for the case-cohort design, a weighted Cox model was applied that was modified according to the Prentice method [18]. We estimated HRs for quartiles of the lipophilic index (quartiles were based on the subcohort distribution) compared with the respective lowest quartile. The use of quartiles was chosen to be able to account for non-linear relationships with a sufficiently high number of incident cases in each category. Cox regression models were first computed with stratification for integers of age and adjustment for sex (model 1), and then with further adjustment for sports activity (0, 0.1–4.0, >4.0 h/week); biking (0, 0.1–2.4, 2.5–4.9, ≥5 h/week); smoking status (never, past, current <20 cigarettes/day, current ≥20 cigarettes/day); education (in or no training, vocational training, technical school or technical college or university degree); alcohol intake (0, 0.1–5.0, 5.1–10.0, 10.1–20.0, 20.1–40.0, >40.0 g/day); total energy intake (continuous); coffee intake (continuous); consumption of sugar-sweetened beverages (continuous); dietary PUFA/SFA ratio (continuous); protein intake (energy-adjusted using residuals from linear regression [19]; continuous) and carbohydrate intake (energy-adjusted using residuals from linear regression [19]; continuous) (model 2). In a third model, further adjustments were made for BMI and waist circumference (both continuous). Categorical variables were entered as binary indicator variables into the models. The significance of linear trends across quartiles was tested by assigning each participant the median value for the quartile and modelling this value as a value of a continuous variable. A test for statistical interaction of the lipophilic index with sex was conducted by including a cross-product term in the model.

Multiple linear regression models were used to calculate adjusted means of biomarkers for quartiles of the lipophilic index in the subcohort, applying model 3 adjustments. Due to differing biomarker distributions, these analyses were carried out for men and women separately. We used geometric means for biomarkers that were not normally distributed (triacylglycerol, adiponectin, GGT, CRP, random glucose, HbA_1c_) and arithmetic means if normal distribution was met (HDL-cholesterol, fetuin-A).

We performed several sensitivity analyses to test the robustness of our findings. First, we excluded participants with treated hypertension at baseline (*n* = 389) because it has been shown that membrane fluidity is altered in hypertension [20]. To investigate the possibility of reverse causation, we excluded participants with baseline HbA_1c_ ≥6.5% (47.5 mmol/mol; *n* = 166) in a second sensitivity analysis and incident cases diagnosed with diabetes within the first 2 years of follow-up (*n* = 81) in a third analysis. Fourth, we also investigated whether excluding participants in the unfasted state at blood collection affects the association between the lipophilic index and triacylglycerol levels (*n* = 1,029). Fifth, we repeated analyses without excluding participants with a history of cardiovascular disease or cancer at baseline (*n* = 275, leading to a final *n* of 2,015 for analyses). For each of these sensitivity analyses, model 3 adjustments were applied.

All statistical analyses were performed with SAS (version 9.2, Enterprise Guide 6.1; SAS Institute, Cary, NC, USA). All statistical tests were two-sided and *p* values <0.05 were considered statistically significant.

## Results

Median subcohort proportions of the individual erythrocyte membrane FAs as well as Spearman correlation coefficients of the individual FAs with the lipophilic index are presented in Table 1. The highest proportions in erythrocyte membranes were observed for 16:0 (22.3%), 18:0 (13.8%), 18:1 *n*-9 (12.7%), 20:4 *n*-6 (13.3%) and 18:2 *n*-6 (10.8%). As expected, SFAs generally showed positive correlations with the lipophilic index (exception: 18:0), whereas PUFAs were inversely correlated with the index (exception: 18:3 *n*-6). Correlation coefficients for MUFAs and *trans*-FAs were usually close to zero.

**Table 1:** Melting points of FAs and median proportions of erythrocyte FAs, and correlation coefficients of erythrocyte FAs with lipophilic index for the subcohort of the EPIC-Potsdam study (*n* = 1,406)

FA	Melting point (°C)^a^	Median proportion^b^ (IQR)	Correlation with index (Spearman)
SFAs			
14:0	53.9	0.38 (0.30, 0.47)	0.34****
15:0	52.3	0.21 (0.17, 0.26)	0.27****
16:0	63.1	22.3 (21.2, 23.4)	0.49****
17:0	61.3	0.32 (0.29, 0.36)	0.16****
18:0	69.6	13.8 (12.8, 14.5)	−0.09*
20:0	76.8	0.39 (0.34, 0.44)	0.50****
21:0	74.3	0.04 (0.03, 0.05)	0.22****
22:0	81.5	1.53 (1.32, 1.78)	0.62****
23:0	79.1	0.27 (0.23, 0.32)	0.36****
24:0	87.8	4.1 (3.5, 4.7)	0.70****
MUFAs			
16:1 *n*-7	0	0.44 (0.35, 0.55)	0.16****
18:1 *n*-7	15.0	1.0 (0.9, 1.1)	−0.08*
18:1 *n*-9	16.0	12.7 (12.0, 13.4)	0.08*
20:1 *n*-9	23.3	0.28 (0.25, 0.32)	−0.13****
22:1 *n*-9	34.7	0.29 (0.21, 0.45)	0.08*
24:1 *n*-9	42.8	4.0 (3.5, 4.6)	0.59****
PUFAs			
18:3 *n*-3	−11.2	0.15 (0.13, 0.18)	−0.14****
20:5 *n*-3	−54.1	0.77 (0.61, 0.94)	−0.40****
22:5 *n*-3	−54.1	2.4 (2.1, 2.6)	−0.55****
22:6 *n*-3	−44.2	4.8 (4.1, 5.4)	−0.62****
18:2 *n*-6	−5.0	10.8 (9.9, 11.7)	−0.03
18:3 *n*-6	−11.2	0.05 (0.04, 0.07)	0.15****
20:4 *n*-6	−49.5	13.3 (12.3, 14.2)	−0.71****
*trans-*FAs			
16:1 *n*-7t	31.0	0.17 (0.14, 0.21)	−0.08*
18:1 *n*-9t + 18:1 *n*-7t	44.8	0.51 (0.44, 0.59)	0.12****

^a^Values obtained from the LipidBank database (http://lipidbank.jp/, accessed 19 November 2013)

^b^% of total FAs

*p < 0.05

****p < 0.0001

Table 2 shows baseline characteristics of the subcohort members by quartiles of the lipophilic index. Participants with a high lipophilic index were more likely to be men and had a slightly higher BMI compared with participants with a low index, although this association did not reach statistical significance. Leisure-time physical activity, smoking status and educational attainment were not significantly associated with the lipophilic index. With regard to dietary factors, a high lipophilic index was associated with obtaining a slightly higher proportion of energy from fat at the expense of protein or carbohydrates.

**Table 2:** Baseline characteristics by quartiles of the lipophilic index for the subcohort of the EPIC-Potsdam study (*n* = 1,406)

	Quartile of lipophilic index				*p* value
	1	2	3	4	
*n* (subcohort)	351	352	351	352	
General characteristics					
Age (years)	47.9	47.5	48.6	48.9	0.16
Men (%)	31.3	35.8	39.3	42.3	0.02
BMI (kg/m^2^)	25.1	25.2	25.5	25.6	0.13
WC^a^					
Men	91.0	92.8	91.8	93.6	0.31
Women	78.0	78.0	78.5	78.0	0.43
Sport activities and biking (h/week)	2.0	1.5	2.0	2.0	0.99
Never smokers (%)	49.9	45.7	45.3	46.3	0.45
College/university (%)	35.3	39.2	39.3	40.9	0.39
Dietary intake					
Fat (% energy)	38.5	39.2	39.1	40.3	0.001
Carbohydrates (% energy)	42.2	42.6	42.7	41.2	0.05
Protein (% energy)	13.8	13.9	13.6	13.7	0.03
PUFA/SFA ratio	0.42	0.43	0.42	0.43	0.74
Alcohol (g/day)	9.2	8.6	8.9	9.2	0.82
Coffee (g/day)	302	302	450	450	0.16

Data are medians, unless otherwise indicated

^a^WC, waist circumference

HRs for the association of quartiles of the lipophilic index with diabetes incidence are presented in Table 3. In the age- and sex-controlled model (model 1), we observed a higher diabetes risk when comparing top with bottom quartile of the lipophilic index (HR 1.76 [95% CI 1.25, 2.47], *p* for trend <0.001). This association slightly strengthened after further adjustment for lifestyle factors, education and dietary factors (HR 1.85 [95% CI 1.29, 2.65], *p* for trend <0.001). The HR was moderately attenuated after further adjustment for BMI and waist circumference (model 3), but still statistically significant (HR 1.59 [95% CI 1.08, 2.34], *p* = 0.005). Additional adjustment for diabetes-related FAs did not weaken this association (HR 1.90 [95% CI 1.25, 2.90], *p*<0.001). In our sensitivity analyses, the exclusion of participants with treated hypertension at baseline, baseline HbA_1c_ ≥6.5% (47.5 mmol/mol) or incident cases diagnosed with diabetes within the first 2 years of follow-up did not materially change the results. The inclusion of participants with a history of cardiovascular disease or cancer at baseline did moderately attenuate the HRs (HR comparing top with bottom quartile 1.34 [95% CI 0.96, 1.87]), however, there was still a significant positive trend across the quartiles (*p* = 0.02). The test for interaction of the lipophilic index with sex was not significant (*p* = 0.46).

**Table 3:** Risk of type 2 diabetes by quartiles of the lipophilic index in a case-cohort study embedded in the EPIC-Potsdam cohort (*n* = 1,740)

	Quartile of lipophilic index				
	1	2	3	4	*p* value
Median index	21.5	23.2	24.7	26.8	
*n* (cases/ subcohort)	68/351	70/352	88/351	136/352	
Model 1^a^	1.00 (ref.)	0.97 (0.67, 1.40)	1.28 (0.89, 1.84)	1.76 (1.25, 2.47)	<0.001
Model 2^b^	1.00 (ref.)	0.97 (0.65, 1.43)	1.36 (0.93, 1.99)	1.85 (1.29, 2.65)	<0.001
Model 3^c^	1.00 (ref.)	0.94 (0.61, 1.43)	1.18 (0.78, 1.78)	1.59 (1.08, 2.34)	0.005
Model 3 + diabetes-related FAs^d^	1.00 (ref.)	0.96 (0.62, 1.49)	1.36 (0.88, 2.08)	1.90 (1.25, 2.90)	<0.001

Data are HRs (95% CI)

^a^Model 1 is stratified by age and adjusted for sex

^b^Model 2 is further adjusted for sports activity, biking, smoking status, education, alcohol consumption, total energy intake, coffee intake, sugar-sweetened beverage intake, dietary PUFA/SFA ratio and intake of protein and carbohydrates (energy-adjusted)

^c^Model 3 is further adjusted for BMI and waist circumference

^d^Diabetes-related FAs: 15:0, 17:0, 18:0, 16:1 *n*-7, 18:2 *n*-6, 18:3 *n*-6, and 20:3 *n*-6

In further analyses, we investigated whether the association of the lipophilic index with diabetes risk could be explained by individual FAs, for which a relation to diabetes risk has already been shown. However, we did not observe a substantial attenuation of the HR in our model 3 after additional adjustment for proportions of 15:0 (HR comparing top with bottom quartile of lipophilic index 1.77 [95% CI 1.18, 2.65]), 17:0 (HR 1.85 [95% CI 1.24, 2.76]), 18:0 (HR 1.59 [95% CI 1.08, 2.34]), 16:1 *n*-7 (HR 1.44 [95% CI 0.97, 2.13]), 18:2 *n*-6 (HR 1.58 [95% CI 1.07, 2.33]), 18:3 *n*-6 (HR 1.51 [95% CI 1.02, 2.23]), 20:3 *n*-6 (HR 1.81 [95% CI 1.22, 2.67]), or total SFAs (HR 3.36 [95% CI 1.68, 6.75]), total *trans*-FAs (HR 1.55 [95% CI 1.05, 2.30]), total MUFAs (HR 1.31 [95% CI 0.79, 2.18]) and total PUFAs (HR 2.27 [95% CI 1.12, 4.60]).

ESM Figures 1 and 2 depict mean baseline biomarker levels according to quartiles of the lipophilic index for men and women of the subcohort. In general, we did not observe strong associations of the lipophilic index with the biomarkers. After multivariable adjustment, including body size (model 3), there was a positive association of the lipophilic index with fetuin-A in men (*p* for trend across quartiles <0.001) and a slight positive association with HDL-cholesterol in women (*p* = 0.002). Baseline levels of triacylglycerol, adiponectin, CRP and GGT showed no significant trend across quartiles in both men and women. Excluding participants with treated hypertension at baseline or with baseline HbA_1c_ ≥6.5% (47.5 mmol/mol) did not substantially change these findings. Furthermore, the association between the lipophilic index and triacylglycerol remained materially unchanged after the exclusion of participants in the unfasted state at blood collection (results not shown).

ESM Figures 3 and 4 show plasma random glucose and HbA_1c_ values by quartiles of the lipophilic index for men and women of the subcohort. In women, we detected a positive association of random glucose with the lipophilic index (*p* for trend = 0.01), whereas a slight inverse association was observed for HbA_1c_ (*p* for trend = 0.01). In men, the positive association of the lipophilic index with random glucose was only borderline significant (*p* for trend = 0.05) and the association with HbA_1c_ did not reach statistical significance (*p* for trend = 0.08).

## Discussion

In this prospective study of middle-aged men and women, a high lipophilic index, indicating lesser fluidity of erythrocyte membranes, was associated with a higher risk of developing type 2 diabetes after multivariable adjustments, including body size.

The lipophilic index has been proposed recently as a measure of the FA fluidity of biological samples [11, 12]. It is determined as the mean of the melting points of the individual FAs weighted by their concentration, and hence can easily be applied in large-scale epidemiological studies subject to the availability of FA profile data in biological samples. In our study, we were able to investigate the lipophilic index calculated from the FA profile of actual membranes, namely erythrocyte membranes. Although the FA composition of erythrocyte membranes is not identical compared with other cells important for glucose metabolism such as hepatocytes or muscle cells, membranes of different cell types share a common feature, which lies in the exchange of phospholipids with the plasma phospholipid pool. Nevertheless, it should be noted that membrane fluidity is not only determined by its FA composition, but also by other factors such as the cholesterol content of the membrane, the degree of phospholipid methylation and calcium binding [21–23].

To our knowledge, this is the first study that investigated the lipophilic index as a measure of FA fluidity in relation to type 2 diabetes risk. We observed a higher diabetes risk for participants with a high lipophilic index reflecting lesser fluidity of the erythrocyte membranes. This finding is in line with the long-held notion that membrane fluidity is an important mediator that links intake and metabolism of FAs with diabetes risk. In cell culture studies, it was shown that membrane fluidity affects the glucose transport across membranes as well as the properties of the insulin receptor [8–10], which is in agreement with our findings.

Two earlier epidemiological studies have investigated the lipophilic index in relation to myocardial infarction and CHD [11, 12]. In a matched case–control study from Costa Rica, the lipophilic index for adipose tissue was significantly positively associated with myocardial infarction in multivariable adjusted models [12]. Similarly, the lipophilic index of plasma phospholipids was significantly associated with a higher risk of CHD in the Health Professionals Follow-up Study. The erythrocyte lipophilic index, however, showed no significant association with CHD risk in this study, although there was a tendency towards a positive relation. The authors speculated that higher measurement error for the FA measurements in erythrocytes compared with plasma phospholipids in their study may have led to an attenuation of the statistical association [11].

In addition to the investigation of the biological meaning of the lipophilic index, it is of interest whether the index is an independent predictor of diabetes risk. After multivariable adjustments, including body size measures, the effect size of the association between lipophilic index and diabetes risk was at least as strong in our study as those for a number of individual FAs, which are considered as established diabetes risk markers including 15:0, 17:0, 18:0, 18:2 *n*-6 and 20:3 *n*-6 (see our earlier publication [3] for details on results). Only 16:1 *n*-7 and 18:3 *n*-6 showed a somewhat stronger association according to our earlier quintile analysis [3]. Of note, adjustment for these FAs individually and simultaneously did not lead to a substantial attenuation of the HR for the association between the lipophilic index and diabetes risk, indicating that the lipophilic index may provide predictive value beyond individual FAs with regard to diabetes risk. Similarly, adjustment for total SFAs, total *trans*-FAs and total PUFAs did not lead to an attenuation of the HR between the lipophilic index and diabetes risk, and adjustment for total MUFAs slightly attenuated the HR likely reflecting that some MUFAs showed associations with risk of diabetes (see our earlier publication [3]). As expected, we have observed positive correlations of individual SFAs and negative correlations of PUFAs with the lipophilic index. Among those individual FAs with the strongest correlation with the lipophilic index, the long-chain SFA 24:0 showed a strong positive association with diabetes risk, whereas the PUFAs 20:5 *n*–3, 22:5 *n*–3 and 20:4 *n*–6 tended to be inversely associated with risk in our study, although not significantly [3]. Interestingly, adjustment for these individual FAs also did not attenuate the effect estimates for the association of the lipophilic index with diabetes risk in our study (results not shown), which further corroborates the hypothesis that the lipophilic index is an independent predictor of diabetes incidence.

We observed only very slight, if any, associations of our lipophilic index with baseline levels of metabolic biomarkers. Similar observations were made in the Health Professionals Follow-up Study for the erythrocyte lipophilic index [11]. As FA proportions and metabolic biomarkers have been assessed in a cross-sectional manner (using baseline blood samples), it was not possible to establish the correct time sequence (i.e. FA proportions precede levels of metabolic markers) in this kind of analysis. Still, clear positive cross-sectional associations of the plasma and adipose tissue lipophilic index with triacylglycerol have been detected in earlier studies, whereas less consistent findings were obtained for inflammatory markers [11, 12]. Surprisingly, we observed higher HDL-cholesterol concentrations for women with a high lipophilic index, which is counterintuitive. This finding is in contrast to earlier studies that detected inverse associations of the plasma and dietary lipophilic index with HDL-cholesterol [11, 12]. However, no significant relations were seen for the erythrocyte and adipose tissue lipophilic index in these studies [11, 12]. Given these mixed findings, further studies should be performed to evaluate this relationship, preferably with a prospective design.

Changed insulin sensitivity is a biological mechanism that may link membrane fluidity to diabetes risk. Unfortunately, we could not perform analyses with specific indicators of insulin sensitivity, such as the HOMA index, owing to the small number of fasted participants in our study. Instead, we have performed analyses with other measures of glucose metabolism, namely random glucose and HbA_1c_, which are, however, not specific indicators of insulin sensitivity. The results for the cross-sectional association of the lipophilic index with random glucose and HbA_1c_ were mixed. However, when accounting for the correct time sequence in our prospective analyses, there was a relatively strong, significant, positive relationship between the lipophilic index and diabetes incidence. This relationship turned out to be robust in various sensitivity analyses, suggesting that changed membrane fluidity is an important factor that precedes the development of type 2 diabetes.

Our analysis has several limitations that should be discussed. The lipophilic index is not a direct measurement of cell membrane fluidity. Although the FA composition is a strong determinant of membrane fluidity, other factors also play a role [21–23]. Further studies using direct methods to determine membrane fluidity are warranted to confirm our findings. Furthermore, melting points of some FAs, especially those with very low proportions in biological tissues (see Methods), were not available in the LipidBank database and hence could not be considered in the calculation of the lipophilic index. However, our results remained substantially the same in a sensitivity analysis for which we excluded specific FAs (proportion of less than 0.5% of total FAs) from the lipophilic index (data not shown). We considered only clinically apparent type 2 diabetes and did not screen our study population for diabetes at baseline, thus it is possible that prevalent but undiagnosed cases remained in our analyses. Still, excluding participants with a baseline HbA_1c_ higher than 6.5% (47.5 mmol/mol) or incident cases diagnosed with diabetes within the first 2 years of follow-up did not appreciably change our results, suggesting that reverse causation should not have had major effects on our findings. Further, we did not screen participants for incident diabetes during follow-up. However, all self-reports of diabetes during follow-up were verified through the treating physician in our study. Given the resulting high specificity and positive predictive value of the disease classification, the remaining misclassification (unidentified cases) should not have biased the estimated risk [24]. Strengths of our study include the wide profile of FAs (*n* = 25 individual FAs) considered in the calculation of the lipophilic index as well as the use of actual membranes to reflect FA fluidity. The prospective design and high rate of follow-up made reverse causation and bias through loss to follow-up less likely. Comprehensive data on diet, lifestyle and other risk factors allowed us to account for potential confounders in detail.

In conclusion, our data suggest that a high lipophilic index reflecting a lower fluidity of erythrocyte membranes is associated with a higher risk of type 2 diabetes. Our findings corroborate the hypothesis that membrane fluidity may be an important mediator that links intake and metabolism of FAs to diabetes risk. Hence, interventions aiming at an improvement of cell membrane fluidity may have the potential to lower diabetes risk. Our findings also suggest that the lipophilic index is of value as an independent predictor of the incidence of type 2 diabetes.

## Electronic supplementary material

Below is the link to the electronic supplementary material.

ESM Methods (PDF 72 kb)

ESM Fig. 1 (PDF 18 kb)

ESM Fig. 2 (PDF 17 kb)

ESM Fig. 3 (PDF 8 kb)

ESM Fig. 4 (PDF 8 kb)

## Abbreviations

CRP: C-reactive protein
EPIC: European Prospective Investigation into Cancer and Nutrition
FA: Fatty acid
FAME: Fatty acid methyl ester
GGT: Gamma-glutamyl transferase
PUFA: Polyunsaturated fatty acid
SFA: Saturated fatty acid
