# Supplementary material for: Erythrocyte membrane fatty acid fluidity and risk of type 2 diabetes in the EPIC-Potsdam study
Source: Diabetologia. 2014 Oct 25;58(2):282–9. doi: 10.1007/s00125-014-3421-7 (PMC4287658; doi:10.1007/s00125-014-3421-7)
Supplement: Supplementary file 1 — (PDF 72 kb) [file 125_2014_3421_MOESM1_ESM.pdf]

ESM Methods

Ascertainment of type 2 diabetes in the EPIC-Potsdam study

The prevalence of diabetes mellitus at baseline was evaluated by a physician using information on self-reported medical diagnoses, medication records and dieting behavior. Uncertainties regarding a proper diagnosis at baseline were clarified with the participant or treating physician.

Incident cases of diabetes were identified during follow-up via self-reports of a diabetes diagnosis, diabetes-relevant medication or dietary treatment due to diabetes. All incident cases were verified by questionnaires mailed to the diagnosing physician asking about the date and type of diagnosis, diagnostic tests and treatment of diabetes. Only cases with a physician diagnosis of type 2 diabetes (ICD10: E11) and a diagnosis date after the baseline examination were considered as confirmed incident cases of type 2 diabetes.

Measurement of fatty acid composition in erythrocytes

Erythrocyte membrane fatty acids were measured between February and June 2008. To a sample of 200 µL erythrocytes, 800 µL distilled water was added and centrifuged for 10 min at 3000 rpm. The pellet, which contained the phospholipid membranes, was washed with 800 µL distilled water and centrifuged again. After the addition of 400 µL distilled water and 3 mL chloroform/methanol (1:1), the mixture was shaken. The chloroform layer was transferred into another tube, and the solvent was removed by evaporation. The phospholipids were hydrolysed and methylated simultaneously with a mixture of 100 µL toluene and 0.5 mL BF_3/MeOH for 60 min at 100°C in a heating block. After cooling, 800 µL distilled water and 800 µL hexane were added. After shaking and settling, the hexane layer (upper layer) that

contained the methylated fatty acids (FAME) was transferred to gas chromatography vials and stored at -20°C until analysis. The FAME were separated on a 100-m x 0.25-mm internal diameter wall-coated with a 0.25 µm of CP-Select chemically bonded (Varian Inc, Middelburg, Netherlands) with a GC-3900 gas chromatograph equipped with a CP 8400 autoinjector (Varian Inc, Middelburg, Netherlands). The Galaxie software version 1.9.3.2. (Varian Inc) was used for quantification and identification of peaks. A baseline separation of over 50 FAME peaks was accomplished by means of mixed FAME standards (Sigma, Zwijndrecht, Netherlands). The analytic conditions used were as follows: a volume injected of 1 µL carrier gas nitrogen (1.1 mL/min), injector temperature at 250°C, flame ionization detection at 275°C, split ratio of 1:20 and an oven temperature from 185°C to 245°C with a stepped temperature program within a total run time of 57 min. The fatty acids were expressed as the percentage of total fatty acids present in the chromatogram. Intra-assay coefficients of variation calculated from a total number of 40 fatty acid measurements in a subset of 20 samples were ≤10% for most fatty acids considered in the statistical analysis, with the exception of 16:1n-9 (16.0%), 16:3n-4 (12.5%), 18:3n-6 (18.7%) and 21:0 (12.6%).

Definition of unreliable measurements

The following exclusions have been applied based on fatty acid values:

-Samples from batches with three times higher median or range for 15:0 proportions compared to other batches, which is likely to indicate an overlap of the 15:0 peak with the peak of another substance in the chromatogram (*n*=105 for cases, *n*=216 for subcohort).

-Samples from batches with median values outside the interquartile range from other batches for certain polyunsaturated fatty acids, namely 22:6n-3, 20:3n-6, 20:4n-6 and 22:4n-6 (*n*=208 for cases, *n*=382 for subcohort).

The following exclusions have been applied based on biomarker values:

-Missing values for biomarkers (*n*=10 for cases, *n*=25 for subcohort)

-Insufficiently filled blood monovettes leading to an unknown dilution by citrate (*n*=26 for cases, *n*=77 for subcohort)

-Fetuin-A concentration < 0.0625 g/L (*n*=0 for cases, *n*=1 for subcohort)

-GGT activity ≤ 0 U/L (*n*=0 for cases, *n*=3 for subcohort)

-Triacylglycerol concentration < 10 mg/dl (*n*=0 for cases, *n*= 3 for subcohort)

-HDL cholesterol concentration > total cholesterol concentration (*n*=0 for cases, *n*=8 for subcohort)

Other measurements

All participants were asked to complete a semi-quantitative food frequency questionnaire (FFQ). This FFQ assessed the average intake frequency and portion size of 148 foods consumed during the 12 months prior to examination. Intake frequency was measured with ten categories, ranging from 'never' to 'five times per day or more'. Portion sizes were estimated using photographs of standard portion sizes. Information on intake frequency and portion size was used to calculate the amount of each food item in grams consumed on average per day. Nutrient intake was calculated from the food items according to the German Food Code and Nutrient Data Base [1] version II.3. The validity and reproducibility of the FFQ have been described previously [2-4]. Information on education, smoking and leisure time and occupational physical activity were assessed at baseline with a self-administered questionnaire and a personal computer-guided interview. We considered sports activities and biking as leisure time physical activities, both calculated as the average time spent per week

during the twelve months before baseline recruitment. Anthropometric measurement procedures followed standard protocols under strict quality control [5, 6].

References

[1] Dehne LI, Klemm C, Henseler G, Hermann-Kunz E (1999) The German Food Code and Nutrient Data Base (BLS II.2). Eur J Epidemiol 15: 355-359

[2] Bohlscheid-Thomas S, Hoting I, Boeing H, Wahrendorf J (1997) Reproducibility and relative validity of energy and macronutrient intake of a food frequency questionnaire developed for the German part of the EPIC project. European Prospective Investigation into Cancer and Nutrition. Int J Epidemiol 26 Suppl 1: S71-81

[3] Bohlscheid-Thomas S, Hoting I, Boeing H, Wahrendorf J (1997) Reproducibility and relative validity of food group intake in a food frequency questionnaire developed for the German part of the EPIC project. European Prospective Investigation into Cancer and Nutrition. Int J Epidemiol 26 Suppl 1: S59-70

[4] Kroke A, Klipstein-Grobusch K, Voss S, et al. (1999) Validation of a self-administered food-frequency questionnaire administered in the European Prospective Investigation into Cancer and Nutrition (EPIC) Study: comparison of energy, protein, and macronutrient intakes estimated with the doubly labeled water, urinary nitrogen, and repeated 24-h dietary recall methods. Am J Clin Nutr 70: 439-447

[5] Kroke A, Bergmann MM, Lotze G, Jeckel A, Klipstein-Grobusch K, Boeing H (1999) Measures of quality control in the German component of the EPIC study. European Prospective Investigation into Cancer and Nutrition. Ann Nutr Metab 43: 216-224

[6] Klipstein-Grobusch K, Georg T, Boeing H (1997) Interviewer variability in anthropometric measurements and estimates of body composition. Int J Epidemiol 26 Suppl 1: S174-180
