# Supplementary material for: Erythrocyte membrane fatty acid fluidity and risk of type 2 diabetes in the EPIC-Potsdam study
Source: Diabetologia. 2014 Oct 25;58(2):282–9. doi: 10.1007/s00125-014-3421-7 (PMC4287658; doi:10.1007/s00125-014-3421-7)
Supplement: Supplementary file 2 — (PDF 18 kb) [file 125_2014_3421_MOESM2_ESM.pdf]

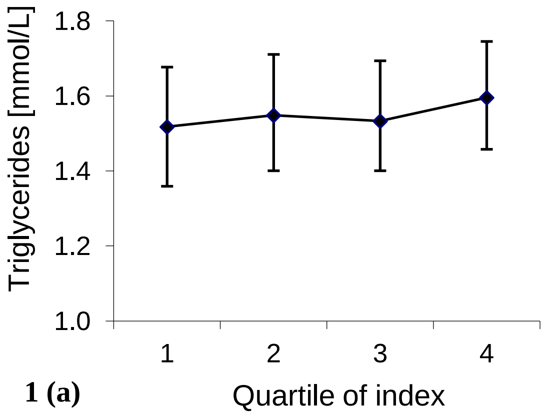

1 (a)

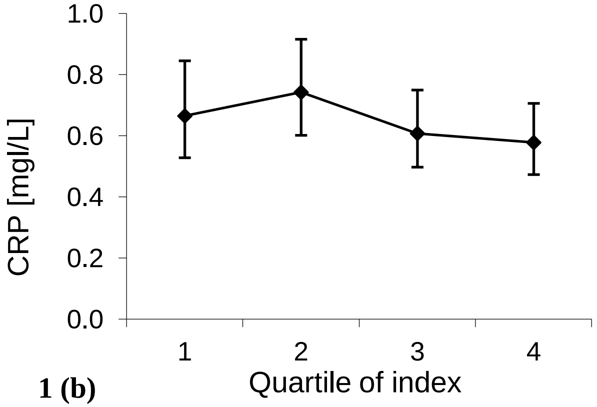

1 (b)

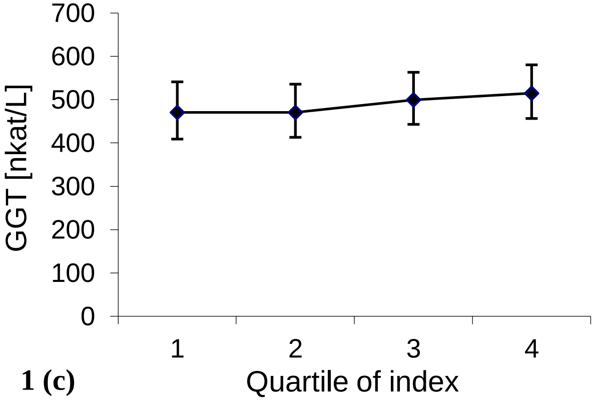

1 (c)

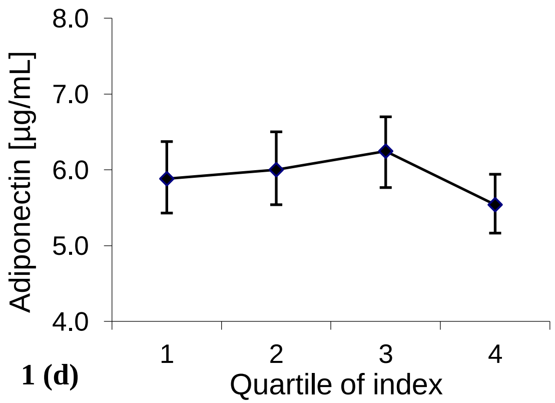

1 (d)

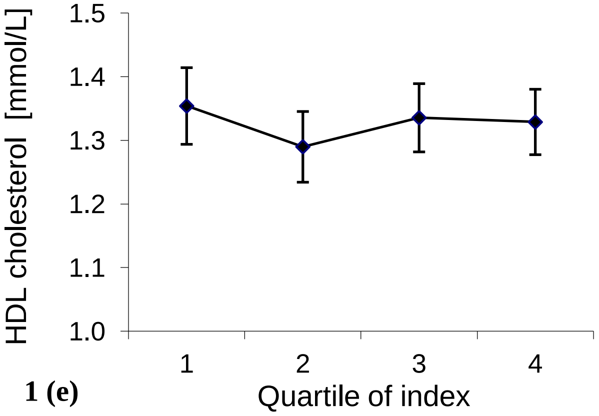

1 (e)

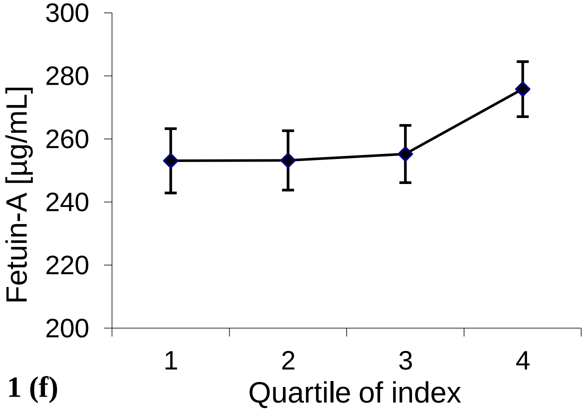

1 (f)

ESM Figure 1: Plasma biomarkers by quartiles of the lipophilic index for men (*n*=523) of the subcohort of the EPIC-Potsdam study

1 (a) Triacylglycerol (p trend=0.47), 1 (b) CRP (p trend=0.23), 1 (c) GGT (p trend=0.25), 1 (d) Adiponectin (p trend=0.24), 1 (e) HDL cholesterol (p trend=0.90), 1 (f) Fetuin-A (p trend<0.001). Values are adjusted geometric (triacylglcerol, adiponectin, CRP, GGT) or arithmetic means (HDL

cholesterol, fetuin-A). Adjustments have been made for age, sports activity, biking, smoking status, education, alcohol consumption, total energy intake, coffee intake, sugar-sweetened beverage intake, dietary PUFA/SFA ratio, intake of protein and carbohydrates (energy-adjusted), BMI and waist circumference.
