# Supplementary material for: Erythrocyte membrane fatty acid fluidity and risk of type 2 diabetes in the EPIC-Potsdam study
Source: Diabetologia. 2014 Oct 25;58(2):282–9. doi: 10.1007/s00125-014-3421-7 (PMC4287658; doi:10.1007/s00125-014-3421-7)
Supplement: Supplementary file 3 — (PDF 17 kb) [file 125_2014_3421_MOESM3_ESM.pdf]

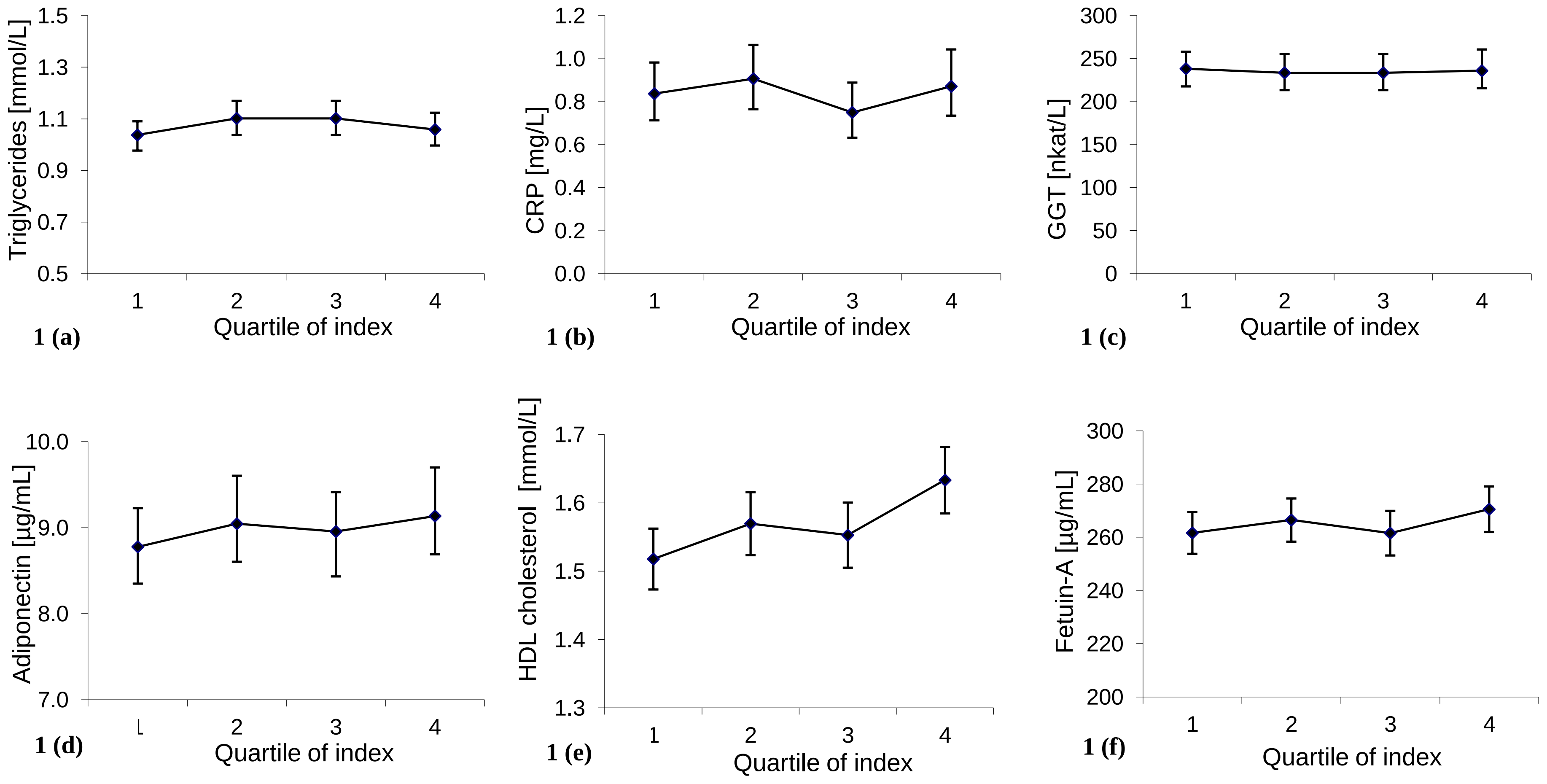

ESM Figure 2: Plasma biomarkers by quartiles of the lipophilic index for women (*n*=883) of the subcohort of the EPIC-Potsdam study

2 a) Triacylglycerol (p trend=0.64), 2 b) CRP (p trend=0.93), 2 c) GGT (p trend=0.98), 2 d) Adiponectin (p trend=0.36), 2 e) HDL cholesterol (p trend=0.002), 2 f) Fetuin-A (p trend=0.22). Values are adjusted geometric (triacylglcerol, adiponectin, CRP, GGT) or arithmetic means (HDL

cholesterol, fetuin-A). Adjustments have been made for age, sports activity, biking, smoking status, education, alcohol consumption, total energy intake, coffee intake, sugar-sweetened beverage intake, dietary PUFA/SFA ratio, intake of protein and carbohydrates (energy-adjusted), BMI and waist circumference.
