# Supplementary material for: Erythrocyte membrane fatty acid fluidity and risk of type 2 diabetes in the EPIC-Potsdam study
Source: Diabetologia. 2014 Oct 25;58(2):282–9. doi: 10.1007/s00125-014-3421-7 (PMC4287658; doi:10.1007/s00125-014-3421-7)
Supplement: Supplementary file 4 — (PDF 8 kb) [file 125_2014_3421_MOESM4_ESM.pdf]

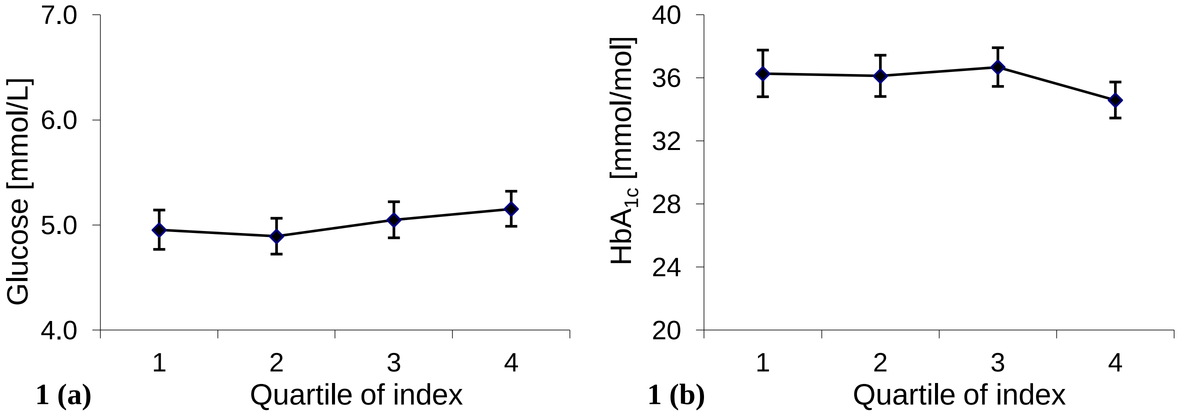

1 (a) **1 (b)**

ESM Figure 3: Measures of glucose metabolism by quartiles of the lipophilic index for men of the subcohort of the EPIC-Potsdam study

1 (a) Plasma random glucose (p trend=0.05), 1 (b) HbA_{1c} (p trend=0.08). Values are adjusted geometric means. Adjustments have been made for age, sports activity, biking, smoking status, education, alcohol consumption, total energy intake, coffee intake, sugar-sweetened beverage intake, dietary PUFA/SFA ratio, intake of protein and carbohydrates (energy-adjusted), BMI and waist circumference.
